# Wood vinegar for sheep receiving high-concentrate diets

**DOI:** 10.5194/aab-68-619-2025

**Published:** 2025-10-22

**Authors:** Vitor L. de L. Melo, Thiago L. A. C. de Araújo, Patrícia de O. Lima, Salenilda S. Firmino, Pedro P. N. de Oliveira, Alexandre S. Pimenta, Michelly F. Macedo, José G. L. de Almeida, Antônia V. de A. F. Amâncio, Maiko R. T. Dantas, Dorgival M. de Lima Júnior

**Affiliations:** 1 Department of Animal Sciences, Universidade Federal Rural do Semi-Árido, Francisco Mota Street, 572, Costa e Silva, 59.625-900, Mossoró-RN, Brazil; 2 Specialized Academic Unit in Agricultural Sciences, Universidade Federal do Rio Grande do Norte, Highway 160-Km 03, Jundiaí, 59280-000, Macaíba-RN, Brazil

## Abstract

The antibacterial, antifungal, and anti-inflammatory properties of refined wood vinegar make it a promising product for ruminant nutrition. This study aimed to evaluate the effect of increasing oral doses (0, 10, 20, 30, and 40 mL d^−1^) of wood vinegar (WV) on the intake, apparent digestibility, ingestive behavior, water balance, ruminal parameters, serum biochemistry, nitrogen balance, and physiology of sheep fed with high levels of concentrate. We used five castrated male sheep, 1/2 Dorper × 1/2 Santa Inês, with an average age of 36 months and an average weight of 59.34±5.73 kg, in a Latin-square design. The animals were provided a total mixed ration formulated at a roughage-to-concentrate ratio of 20:80 , which was offered twice daily following the delivery of half the daily dose of WV. There was no effect ( P>0.05 ) of WV on dry-matter intake. Increasing WV levels linearly increased ( P<0.05 ) the neutral-detergent fiber intake and apparent digestibility of crude protein in the sheep's diet. Feeding time was increased ( P<0.05 ) by increasing the supply of WV to the sheep; pH values decreased ( P<0.05 ), and ruminal ammonia nitrogen increased ( P<0.05 ) with an increasing dose of WV. The increase in the WV supplied did not influence the water absorbed and nitrogen retained by the lambs ( P>0.05 ). The supply of WV to lambs altered the concentrations of total protein, globulin, urea, and gamma-glutamyltransferase ( P<0.05 ). It may be advisable to offer up to 40 mL d^−1^ of WV to sheep fed high-concentrate diets.

## 1 Introduction

The confinement of sheep improves the weight gain and carcass traits of the animals compared to pasture-raised animals (Jiménez et al., 2019). However, feeding ruminants with a high level of grain can increase the production of ruminal fermentation gases and the levels of nitrogen compounds in the excreta (Wang et al., 2018). In addition, metabolic diseases such as acidosis and tympanism limit the performance of confined animals (Montano et al., 2015). In this context, nutritional additives can be used in high-grain diets to reduce negative environmental and animal impacts (Ahmed et al., 2024).

With the European market being closed to animal products from herds fed ionophore additives, there is an increasing interest in natural alternatives that can deliver similar effects to those of ionophores. Essential oils and tannins have been consolidated as alternatives to ionophore antibiotics (Orzuna-Orzuna et al., 2021; Torres et al., 2021; Yanza et al., 2021). However, the evaluation and testing of other sources of bioactive compounds are necessary. Wood vinegar (WV) has been attracting research interest in the areas of agriculture and livestock (Mhamdi, 2023; Pimenta et al., 2018). WV is a liquid byproduct of the controlled burning of lignocellulosic residues and is rich in acetic, phenolic, and carboxylic acids that have bactericidal activity against gram-positive and some gram-negative strains (Araújo et al., 2018; Jankowsky et al., 2018). The bactericidal and/or bacteriostatic action of WV seems to be associated with a change in the pH of the bacterial protoplasm (Araújo et al., 2018), similarly to that of ionophores. WV was inefficient in reducing CH4 (O'Reilly et al., 2021) but decreased the in vitro degradabilities of dry and organic matter and protozoan populations (Qomariyah et al., 2021). When tested in vivo in cattle, WV (3 % and 6 % of the total diet) reduced dry-matter intake and plasma glucose but increased daily weight gain (at the 3 % level), intramuscular fat, and meat tenderness (Kook and Kim, 2003).

WV contains several substances with antimicrobial activity, especially phenols. It is presented as an interesting alternative to synthetic microbials due to its characterization as a natural product with a beneficial effect without any commercial use restriction. Therefore, the use of WV in livestock should be studied further, mainly due to its widely described antibacterial, antifungal, and anti-inflammatory activity effects (Gama et al., 2024; Yıldızlı et al., 2024). We hypothesize that increasing doses of WV supplementation in the diet will positively affect nutrient utilization, rumen fermentation, and physiological responses in sheep fed high-concentrate diets.

## 2 Material and methods

All experimental procedures were performed according to the national animal welfare standards following Opinion 33/2021 of the Ethics Committee on Animal Use (CEUA) of the Universidade Federal Rural do Semi-Árido (UFERSA).

### 2.1 Animals, treatments, and design

The experiment was conducted at UFERSA, Mossoró, Rio Grande do Norte, Brazil (5°11^′^17^′′^ S, 37°20^′^39^′′^ W). The climate of this region is classified as “BSwh” according to the Köppen–Geiger classification, indicating a hot, semi-arid steppe climate characterized by dry winters and an average annual temperature exceeding 18 °C. The average temperature during the experiment was 27.8 °C, and average relative air humidity was 65.4 %.

The WV was purified through vacuum bi-distillation of the crude wood vinegar. The crude extract was obtained from the carbonization of the wood of a hybrid of *Eucalyptus urophylla* and *Eucalyptus grandis* (*Eucalyptus urograndis* – clone GG100). The production and purification procedures and the chemical composition of the wood were described in detail by Pimenta et al. (2023).

We used five castrated male sheep, 1/2 Dorper × 1/2 Santa Inês, with an average age of 36 months and an average body weight (BW) of 59.34±5.73 kg. Before the experiment, the animals were treated with Ripercol^®^ L (Zoetis, USA) against endoparasites and ectoparasites and housed in metabolism cages with a feeder, a drinker, and devices for collecting feces and urine separately. The metabolism cages were installed in a shed covered with clay tile, with a ceiling height of 3.5 m and a concrete floor, and were provided with artificial lighting at night.

Before the experimental period, the animals were adapted to the high-concentrate diet by gradually increasing the proportion of concentrate in the total diet by 20 % every 8 d for 24 d, starting with a roughage : concentrate ratio of 80:20 until reaching a ratio of 20:80 . The experiment lasted 85 d and was divided into five periods of 17 d, of which 12 d were for animal adaptation and 5 d were for data collection. The experiment used a Latin-square design, and the treatments consisted of doses of 0, 10, 20, 30, and 40 mL d^−1^ of WV.

The concentrate consisted of crushed corn grain, soybean meal, wheat bran, mineral salt, calcitic limestone, and sea salt (NaCl) (Table 1). The rations were formulated to meet the maintenance requirements of 60 kg BW male sheep (1.05 kg d^−1^ of dry matter, 137.00 g d^−1^ of crude protein (CP), and 2.02 Mcal d^−1^ of metabolizable energy), as calculated based on the NRC (2007) guidelines. For the roughage, Tifton 85 hay was purchased in bales of 15–18 kg, crushed in a forage machine, adjusted to produce particles of 30 mm, placed in plastic bags, and stored in a dry location. The daily food supply was divided into two meals as fully mixed feed at 08:00 and 16:00 LT (UTC − 3). A 10 % surplus of the previous day's supply was recommended for voluntary intake.

The WV was administered orally using a 50 cc automatic vaccinator with an oral dosing nozzle (model 91, Walmur^®^, Brazil) to ensure the daily supply of the recommended levels of WV. The total dosage of WV was supplied twice, with half the dose being provided at 08:00 LT and the other half being provided at 16:00 LT, immediately after feeding. Animals that did not receive WV were given 15 mL of water as a placebo solution.

**Table 1:** Percentage and chemical composition of the concentrate and chemical composition of the forage and diet used in the experimental diet.

		Concentrate	Diet
Tifton 85 hay (%)			20.00
Corn grain (%)		73.12	58.50
Wheat bran (%)		15.25	12.20
Soybean meal (%)		9.13	7.30
Sea salt (%)		1.00	0.80
Mineral salt^a^ (%)		1.00	0.80
Calcitic limestone (%)		0.50	0.40
Chemical composition	Tifton 85 hay		
(g kg^−1^)	(*Cynodon* spp.)		
Dry matter	915.6	917.4	917.0
Organic matter	930.6	832.3	852.0
Crude protein	95.1	123.5	117.8
Ether extract	17.7	44.5	39.1
NDFap^b^	685.4	104.8	220.9
ADF^c^	335.0	33.9	94.1
ADL^d^	33.2	7.4	12.6
NFC^e^	107.0	671.0	558.2

^a^ Guaranty levels: calcium – 120 g, phosphorus – 87 g, sodium – 147 g, sulfur – 18 g, cobalt – 40 mg, copper – 590 mg, iodine – 80 mg, chrome – 20 mg, manganese – 1300 mg, selenium – 15 mg, zinc – 3800 mg, iron – 1800 mg, molybdenum – 10 mg, fluorine (max) – 870 mg. ^b^ Neutral-detergent fiber corrected for ash and protein. ^c^ Acid detergent fiber. ^d^ Acid detergent lignin. ^e^ Non-fibrous carbohydrates.

Samples of the provisioned diet, leftovers, feces, and urine were collected during the 5 d of data collection to determine the intake of nutrients, apparent digestibility, and nitrogen balance. Blood samples were taken on the fourth day, and ruminal fluid was collected on the fifth day to determine the metabolic parameters.

### 2.2 Nutrient intake and digestibility

The amounts of supplied diet and leftovers were recorded daily to estimate intake. Nutrient intake was calculated as the difference in chemical composition between the samples of feed supplied and the leftovers collected, which were collected, stored in plastic bags, and frozen at -15 °C until analysis. The apparent digestibility of nutrients was determined using the total feces collection method. The feces produced by each animal were collected in plastic trays adapted to the metabolism cage. The contents of the feces collection trays were weighed every 24 h at 07:00 LT, followed by homogenization and sampling of 10 % of the total weight of the fecal sample. The samples were sprayed with a sulfuric-acid solution ( 1:1 ), placed in plastic bags, and stored at -15 °C until analysis.

### 2.3 Analysis of the total phenol content of the wood vinegar

The total phenols in the WV were determined according to the methodology of Meda et al. (2005) using the Folin–Ciocalteau reagent. From the WV, 0.5 mL aliquots of the WV (final concentration of 20 ppm) were separately placed into test tubes; 2.5 mL of Folin–Ciocalteau reagent (0.2 N) was added to each tube; and, after 5 min, 2 mL of sodium carbonate (75 g L^−1^) was added. The tubes were under light for 2 h. Absorbance was measured using a methanol blank in spectrophotometry (model UV-340G, Gehaka^®^, Brazil) at 760 nm. The results were extrapolated in a calibration curve obtained with gallic-acid standard (20–200 ppm) and expressed in grams of gallic-acid equivalent (GAE) per 100 mL of extract. For an advanced analysis of the bi-distilled WV, see Pimenta et al. (2018).

### 2.4 Chemical composition analysis

The samples of diet, leftovers, and feces were thawed for 24 h in a refrigerator (4 °C), homogenized, and pre-dried in forced air ventilation ovens (model TE-394/2-MP, Tecnal^®^, Brazil) at 55 °C for 72 h. After pre-drying, the material was ground in a knife mill (model R-TE-680, Tecnal^®^, Brazil) with a 1 mm sieve and placed in hermetically sealed containers for further chemical composition analysis.

The samples were analyzed for their chemical constituents using the methods described in AOAC (2019) for dry matter (DM) (method no. 934.01); ash (MM) (method no. 942.05); CP (method no. 981.10); ether extract (EE) (method no. 920.39); and neutral-detergent-insoluble fiber (NDF), corrected for ash and protein, and acid detergent fiber according to the methodology described by Van Soest et al. (1991), with adaptations by Mertens (2002) and Licitra et al. (1996). Non-fibrous carbohydrates (NFCs) were calculated according to Detmann and Valadares Filho (2010). The diets' total digestible nutrient contents (TDN) were estimated according to the equation described by Weiss et al. (1992).

### 2.5 Ingestive behavior

Ingestive behavior was analyzed to determine the behavioral variables (idleness, ruminating, eating) on the first day of each collection period. The lambs were observed by a trained evaluator for 24 h (starting at 06:00 LT and ending at 05:55 LT the next day) by instantaneous scanning at intervals of 5 min, and the behavior of the animal at each time point was recorded according to the methodology described by Eustáquio Filho et al. (2016). The number of chews per ruminated bolus (no. bolus^−1^) and the time spent ruminating each bolus (s bolus^−1^), measured by using a digital stopwatch, were determined through direct observation simultaneously with the observations of the behavioral variables. Observations were made of three rumen boluses per animal during three different periods of the day (10:00–12:00, 14:00–16:00, and 18:00–20:00 LT). The feed and rumination efficiencies in DM (g h^−1^) and neutral-detergent-insoluble fiber (g h^−1^) were calculated by dividing the intake by the total time spent in feeding and/or ruminating during 24 h, respectively (Bürger et al., 2000).

### 2.6 Collection of ruminal fluid, pH, and ammonia nitrogen

Ruminal fluid was collected from each animal on the fifth data collection day to determine pH and ammonia nitrogen (N– ${\mathrm{NH}}_{3}$ ) levels. Approximately 100 mL of ruminal fluid was obtained with an esophageal catheter 0, 2, 4, and 6 h after the morning meal. The esophageal probe was coupled to a vacuum pump (model 131, Prismatec^®^, Brazil). The material sucked by the probe was filtered through double cotton gauze. After filtration, the pH was immediately determined using a digital potentiometer (model PG1800, Gehaka^®^, Brazil). Aliquots of 50 mL of fluid were stored in 80 mL polyethylene jars containing 1 mL of sulfuric acid ( 1:1 ) and were frozen at -15 °C for subsequent determination of the concentration of N–NH_3_ by the methodology described by Detmann et al. (2012).

### 2.7 Blood collection and serum biochemistry

Blood samples were collected from each animal by means of jugular-vein puncture on the fourth data collection day of each period, 4 h after the morning feeding, with Vacutainer tubes. The tubes for leukocyte count contained the anticoagulant ethylenediaminetetraacetic acid; the serum biochemistry tubes had no anticoagulant.

The techniques used in the blood count followed the recommendations of Jain (1993). Turk solution ( 1:20 dilution) was used for counting leukocytes with a hemocytometer. The samples without anticoagulant were centrifuged (model SH120, Global^®^, Brazil) at 5000 rpm for 15 min, stored in Eppendorf mini-tubes, and frozen at -15 °C. Subsequently, the mini-tubes were thawed at room temperature and analyzed to determine the concentrations of cholesterol, glucose, albumin, globulin, urea, creatinine, total proteins, aspartate transaminase (AST), gamma-glutamyltransaminase (GGT), and triglycerides by using commercial kits (Labtest^®^, Brazil), with the aid of a semi-automatic biochemical analyzer (model LB-B200, Bioplus, Brazil), as indicated by the manufacturer.

### 2.8 Water balance

The total water intake via drink and food was determined during the 5 d of data collection of each period. Water was supplied in 15 L polyethylene buckets offered in the metabolism cage. Daily water intake was calculated as the difference between the weight of the water supplied and the weight of the remaining water. Water losses by evaporation were determined by weighing two buckets of water strategically placed in the installation to be inaccessible to the sheep. Evaporation data were used to correct water intake.

Dietary water intake was estimated by determining the moisture content of the food and leftovers. The total water excreted was estimated by summing the volume of water excreted in feces and urine. Each animal's urine was collected in plastic trays attached to the metabolism cages during the entire collection period. To prevent nitrogen evaporation, 20 mL of sulfuric acid ( 1:1 ) was added to the trays. All urine was weighed daily, and a sample of 10 % of the total volume was collected, filtered in gauze, packaged in bottles, and frozen at -10 °C until analysis. A urine aliquot was used to determine total nitrogen (AOAC, 2019; method no. 981.10). Absorbed nitrogen (g d^−1^) was obtained based on the difference between ingested nitrogen and excreted nitrogen, while nitrogen retention was obtained based on consumed nitrogen (g d^−1^) minus excreted nitrogen minus nitrogen in urine (g d^−1^).

### 2.9 Nitrogen balance

The animals' urine was collected in plastic trays attached to the metabolism cages during the entire collection period. To prevent nitrogen evaporation, 20 mL of sulfuric acid ( 1:1 ) was added to the trays. All urine was weighed daily, and a sample of 10 % of the total volume was collected, filtered in gauze, packaged in bottles, and frozen at -10 °C until analysis. A urine aliquot was used to determine total nitrogen (AOAC, 2019; method no. 981.10). Absorbed nitrogen (g d^−1^) was obtained based on the difference between ingested and excreted nitrogen in feces, while nitrogen retention was obtained based on consumed nitrogen (g d^−1^) minus nitrogen excreted in feces minus nitrogen excreted in urine (g d^−1^).

### 2.10 Statistics

The data were subjected to an analysis of variance, and the treatment effect unfolded in its linear and quadratic components through polynomial orthogonal contrasts. The dependent variables were analyzed with a 5×5 Latin-square design using the PROC MIXED feature in the SAS On Demand for Academics software as follows:

1 $$Y_{ijk}=\mu_{ijk}+T_{i}+P_{j}+A_{k}+\varepsilon_{ijk},$$

where $Y_{ijk}$ is a continuous dependent response variable, $\mu_{ijk}$ is the overall average, $T_{i}$ is the fixed effect of treatment ( i=0 , 10, 20, 30, or 40 mL), $P_{j}$ is the effect of the period ( j=1 –5), $A_{k}$ is the random effect of the animal within the treatment ( k=1 –5), and $\varepsilon_{ijk}$ is the unidentifiable random error.

When treatment effects were significant, the control diet (0 mL) was compared to the diets with WV by the Dunnett test. A level of 5 % probability for type-I errors was adopted for all procedures.

## 3 Results

The WV tested in this study had 360.8 mg of total phenols (gallic-acid equivalent per 100 mL). Thus, the animals received 36, 72, 108, and 144 mg d^−1^ of total phenols in the 10, 20, 30, and 40 mL d^−1^ treatments, respectively.

Increasing the WV dose did not influence ( P>0.05 ) the intake of DM, organic matter (OM), CP, and NFCs and the total digestible nutrients of sheep. However, increasing the dose of WV increased ( P<0.05 ) the intake of NDF (Table 2).

**Table 2:** Intake and apparent digestibility of nutrients from the diet of sheep fed high-concentrate diets and increasing levels of wood vinegar (WV).

	Wood vinegar (mL d^−1^)					SEM^a^	P value^b^		
	0	10	20	30	40		Treatment	Linear	Quadratic
Intake (g d^−1^)									
Dry matter	716.03	741.18	853.33	828.87	919.66	57.73	0.4370	0.0791	0.9866
Organic matter	675.21	698.17	805.13	780.22	865.78	54.22	0.4417	0.0814	0.9822
Ash	40.58	43.12	48.47	48.87	54.06	4.10	0.4846	0.0853	0.9870
Crude protein	115.55	117.20	141.85	134.35	149.52	9.58	0.3602	0.0698	0.9225
Ether extract	18.87	19.94	23.39	22.56	23.71	1.79	0.5527	0.1361	0.6589
NDFap^c^	160.86	152.26	184.61	177.20	205.61	11.68	0.1869	0.0355	0.5691
NFC^d^	380.21	408.70	455.03	445.93	486.80	32.41	0.5914	0.1304	0.8692
TDN^e^	539.51	538.99	649.76	658.69	705.04	44.45	0.4367	0.0765	0.9774
Apparent digestibility (%)									
Dry-matter	70.99	63.25	76.13	71.61	82.91	3.09	0.1402	0.0627	0.2930
Organic matter	74.18	66.89	78.38	74.17	84.66	2.80	0.1456	0.0713	0.2679
Crude protein	75.27	65.22	81.02	76.04	85.06	2.76	0.0363	0.0318	0.2726
Ether extract	71.11	62.22	78.10	68.14	80.65	2.94	0.0786	0.1099	0.3460
NDF^c^	54.96	56.48	74.06	49.53	72.43	3.47	0.0561	0.1919	0.9786
NFC^d^	82.71	83.11	86.13	79.36	89.95	1.88	0.2442	0.3066	0.3914
TDN^e^	0.74	0.73	0.75	0.80	0.77	0.01	0.3770	0.1477	0.9537

^a^ SEM denotes standard error of the mean. ^b^ P value for linear and quadratic order contrasts. ^c^ Neutral-detergent fiber corrected for ash and protein. ^d^ Non-fibrous carbohydrates. ^e^ Total digestible nutrients.

Increasing the WV dose did not influence ( P>0.05 ) the apparent digestibilities of DM, OM, NDF, and NFCs in the diet. However, an increase in the apparent digestibility of CP was observed with an increase in WV dose. Intake time increased with an increasing WV dose. However, WV dose did not affect rumination time, total chewing time, number of ruminated boluses, or rumination efficiency (Table 3).

**Table 3:** Ingestive behavior of sheep fed high-concentrate diets and increasing levels of wood vinegar (WV).

	Wood vinegar (mL d^−1^)					SEM^a^	P value^b^		
	0	10	20	30	40		Treatment	Linear	Quadratic
Feeding (min d^−1^)	154.00	172.00	186.00	165.00	200.00	11.15	0.0709	0.0267	0.9804
Rumination (min d^−1^)	263.00	300.00	281.00	270.00	299.00	15.49	0.7283	0.5801	0.9286
Leisure (min d^−1^)	1022.00	965.00	972.00	974.00	939.00	20.92	0.3009	0.0769	0.6917
Total chewing (h d^−1^)	417.00	472.00	467.00	435.00	499.00	21.27	0.3088	0.1743	0.9325
Mericic (s bolus^−1^)	49.78	48.09	47.61	45.25	47.92	1.42	0.5957	0.2993	0.3523
Mericic (no. bolus^−1^)	54.71	53.62	54.09	53.18	55.20	2.10	0.9297	0.9309	0.4991
Ruminated boluses (no. d^−1^)	318.40	388.20	354.55	377.13	385.37	25.93	0.5279	0.2451	0.5843
Feed efficiency (kg DM^3^ h^−1^)	0.29	0.25	0.29	0.31	0.25	0.02	0.5569	0.8594	0.6061
Rumination efficiency (kg DM^3^ h^−1^)	0.16	0.16	0.18	0.18	0.18	0.01	0.8166	0.2808	0.8429

^a^ SEM denotes standard error of the mean. ^b^ P value for linear and quadratic order contrasts. ^c^ Dry matter.

Increasing the WV dose did not influence ( P<0.05 ) the amount of water consumed by the animals. There was no effect ( P<0.05 ) of WV dose on the amount of water absorbed or excreted (Table 4).

**Table 4:** Water balance of sheep fed high-concentrate diets and increasing levels of wood vinegar (WV).

	Wood vinegar (mL d^−1^)					SEM^a^	P value^b^		
	0	10	20	30	40		Treatment	Linear	Quadratic
Intake (kg d^−1^)									
Drinking water	2703.60	2935.00	2846.00	3115.60	2962.00	234.25	0.9978	0.9973	0.9991
Water via food	313.51	360.03	311.85	251.73	285.05	16.78	0.1953	0.1190	0.6348
Total water	3017.11	3295.03	3157.85	3367.33	3247.05	237.24	0.5428	0.2845	0.4392
Excretion (kg d^−1^)									
Water via urine	1003.60	874.20	706.40	880.33	862.40	84.40	0.2474	0.3249	0.1063
Water via urine (% of excreted)	83.36	78.27	74.77	83.79	76.92	1.41	0.0242	0.2575	0.2447
Water via feces	191.99	248.11	260.37	194.98	241.48	27.98	0.1418	0.6772	0.7259
Water via faeces (% of excreted)	16.64	21.73	25.23	16.21	23.08	1.41	0.0242	0.2575	0.2447
Total	1195.59	1122.31	966.77	1302.98	1103.88	125.74	1.0000	1.0000	0.9995
Water absorbed	1821.52	2172.71	2191.08	2064.35	2143.18	135.59	0.2380	0.1838	0.1502
Water absorbed (%)	61.68	66.86	70.45	65.33	66.40	1.84	0.1812	0.3000	0.0734

^a^ SEM denotes standard error of the mean. ^b^ P value for linear and quadratic order contrasts.

An increased WV dose did not influence ( P>0.05 ) the amounts of nitrogen consumed, excreted, absorbed, and retained by the sheep (Table 5).

**Table 5:** Nitrogen balance of sheep fed high-concentrate diets and increasing levels of wood vinegar (WV).

	Wood vinegar (mL d^−1^)					SEM^a^	P value^b^		
	0	10	20	30	40		Treatment	Linear	Quadratic
N intake (g d^−1^)	18.49	18.75	22.70	21.50	23.92	1.53	0.3597	0.0697	0.9224
Fecal excretion of N (g d^−1^)	4.12	4.83	4.09	3.64	4.38	0.32	0.4389	0.6321	0.8406
Urinary excretion of N (g d^−1^)	7.41	6.12	6.89	7.69	7.64	0.50	0.5568	0.4276	0.3938
Total excretion of N (g d^−1^)	11.54	10.95	10.98	11.32	14.73	0.85	0.3524	0.1653	0.1511
Total absorption of N (g d^−1^)	10.19	9.46	12.90	10.82	12.25	0.70	0.1202	0.0918	0.7375
Total retention of N (g d^−1^)	2.57	3.03	4.21	3.70	4.36	0.37	0.5274	0.1230	0.6945
Total N retention (% absorbed)	25.62	31.11	40.65	34.74	34.17	2.35	0.1888	0.1328	0.0949
Total N retention (% ingested)	12.00	13.74	21.54	17.48	18.09	1.50	0.1189	0.0655	0.1549

^a^ SEM denotes standard error of the mean. ^b^ P value for linear and quadratic order contrasts.

There was a linear decrease ( P<0.05 ) in ruminal pH and a linear increase ( P<0.05 ) in ruminal N–NH_3_ with increasing WV dose (Table 6). There was an increase ( P<0.05 ) in ruminal pH and N–NH_3_ as a function of post-meal collection time (Table 7).

**Table 6:** Sheep ruminal parameters as a function of increasing levels of wood vinegar (WV).

	Wood vinegar (mL d^−1^)					SEM^a^	P value^b^		
	0	10	20	30	40		Treatment	Linear	Quadratic
pH	7.05	6.93	6.88	6.83	6.83	0.03	0.1048	0.0110	0.3020
N–NH_3_ (mL dL^−1^)	18.41	20.18	19.87	22.67	21.31	0.48	0.1934	0.0479	0.4945

^a^ SEM denotes standard error of the mean. ^b^ P value for linear and quadratic order contrasts.

**Table 7:** Ruminal parameters of sheep as a function of sample collection time (h) after the morning meal.

	Time (h)				SEM^a^	P value^b^		
	0	2	4	6		Time	Linear	Quadratic
pH	6.86	6.83	6.91	7.03	0.03	0.0900	0.0306	0.1831
N–NH_3_ (mL dL^−1^)	19.52	20.08	20.73	21.63	0.48	0.1677	0.0266	0.8088

^a^ SEM denotes standard error of the mean. ^b^ P value for linear and quadratic order contrasts.

**Table 8:** Serum biochemistry of sheep fed high-concentrate diets and increasing levels of wood vinegar (WV).

	Wood vinegar (mL d^−1^)					SEM^a^	P value^b^		
	0	10	20	30	40		Treatment	Linear	Quadratic
Total protein (mg dL^−1^)	5.36	5.30	5.40	5.67	5.93^f^	0.07	0.0158	0.0019	0.1088
Albumin (mg dL^−1^)	3.94	4.03	4.25	4.08	3.76	0.09	0.4256	0.5893	0.0928
Globulin (mg dL^−1^)	1.42	1.26	1.14	1.58	2.24^f^	0.11	0.0027	0.0014	0.0019
Albumin : globulin ratio	2.95	4.07	3.00	2.21	1.77	0.32	0.1669	0.0544	0.2720
Creatinine (mg dL^−1^)	0.97	0.92	0.93	0.92	0.98	0.03	0.4031	0.8218	0.0787
Urea (mg dL^−1^)	44.32	44.05	35.39	36.17	45.45	1.73	0.1741	0.6259	0.0466
AST (IU L^−1^)^c^	135.96	94.35	138.66	107.42	122.29	13.06	0.6607	0.8545	0.6853
GGT (IU L^−1^)^d^	66.01	89.64	80.65	78.31	46.94	5.77	0.0582	0.1220	0.0124
FA (IU L^−1^)^e^	1497.82	1265.33	1029.09	1122.81	1141.89	82.84	0.4587	0.1620	0.2455
Cholesterol (mg dL^−1^)	46.00	58.80	50.80	54.00	51.65	3.89	0.8158	0.7899	0.5115
Triglycerides (mg dL^−1^)	24.54	27.06	27.02	26.71	17.53	2.15	0.5275	0.3276	0.1808
Glucose (mg dL^−1^)	65.88	68.18	72.70	71.60	69.68	1.74	0.1685	0.1236	0.0932

^a^ SEM denotes standard error of the mean. ^b^ P value for linear and quadratic order contrasts. ^c^ Aspartate aminotransferase. ^d^ Gamma-glutamyltransferase. ^e^ Alkaline phosphatase. ^f^ Means that differ from the control (0 mL d^−1^ of WV) according to the Dunnett's test at the level of 5 % probability.

Increasing the WV dose increased ( P<0.05 ) the serum protein levels and influenced ( P<0.05 ) the levels of globulin and urea in a negative quadratic manner. There was a positive quadratic response in GGT levels to an increasing WV dose (Table 8). The dose of WV did not influence glucose, cholesterol, or triglyceride levels ( P>0.05 ).

## 4 Discussion

WV is rich in phenolic compounds such as guaiacol, phenol, cresol, and furfural (Pimenta et al., 2018). These antimicrobial compounds can function as a ruminal fermentation modulator and animal growth promoter (Gama et al., 2024). Additionally, WV is an acidic liquid (pH 2.83). Its supply to animals may stimulate adaptive mechanisms to buffer ruminal pH (González et al., 2012). In this context, we suggest that the observed increase in NDF intake, combined with the increase in WV dose, is associated with the sheep's attempt to regulate ruminal pH since WV may have acidified the ruminal fluid (Moya et al., 2011). Ruminants have chemoreceptors that can detect changes in the chemical characteristics of their ruminal content and stimulate feedback loops, such as increased intake of NDF to favor chewing and salivation, which help control ruminal pH (Clauss and Hummel, 2017). The amount of phenolic compounds consumed by sheep (0.05 0.08 g kg^−1^ DM; 0.13 and 0.16 g kg^−1^ DM) was low according to the meta-analysis of Al Rharad et al. (2025).

Phenolic compounds can alter the ingestive behavior of ruminants (Santos et al., 2021). The increase in food intake time with an increased WV dose can be attributed to the greater intake of roughage – the primary source of NDF – by the animals (Fimbres et al., 2002). Roughage requires a longer chewing period than the bran concentrate to reduce particle size (Mendes et al., 2020). On the other hand, we observed no change in rumination time or the amount of bolus ruminated by the animals, possibly due to there being the same roughage : concentrate ratio in all treatments (Eustáquio Filho et al., 2016). Ahmed et al. (2021) reported an increase in the feeding time of sheep subjected to phytogenic substances in the diet.

Associated with the increased NDF intake is the possibility that there was a reduction in the rate of disappearance of ruminal DM due to the increase in NDF in the ruminal content itself (Goulart et al., 2020). In this context, this reduction in the ruminal DM passage rate may be associated with increased ruminal starch degradation – the primary component of the sheep's diet – and increased energy intake within the rumen (Cui et al., 2019), supporting greater bacterial growth and ruminal microbial protein synthesis (Guo et al., 2021; Zhang et al., 2024) with an increased WV dose. Therefore, the increase in the digestibility of CP with an increased WV dose may be associated with increased synthesis of ruminal microbial protein (Putri et al., 2021), which has greater digestibility and biological value than food protein (Santos et al., 2021).

Increased doses of WV (an acidic liquid) associated with (probable) greater microbial growth – with greater production of organic acids by bacteria – possibly explain the decrease in ruminal pH (Carlis et al., 2021) that we observed in sheep receiving higher doses of WV. In this context, the increase in ruminal N–NH_3_ with an increased WV dose may also be associated with greater microbial growth and its positive effect on the degradation of potentially fermentable OM, mainly degradable protein in sheep's rumen (Shen et al., 2023).

Despite the increase in the apparent digestibility of CP and ruminal N–NH_3_, we observed no effect of WV dose on the excretion, absorption, and retention of nitrogen. We did not expect these results since the effect of phenolic compounds as modulators of nitrogen absorption and retention in the body of animals is well documented (Ahmed et al., 2024). We suggest that the type of diet used (high-concentrate) in all treatments may have interfered with the mechanisms inherent to the sheep's nitrogen balance, such as nitrogen recycling, serum protein production, and liver transaminases (Abdoun et al., 2006; Bach et al., 2005).

The increase in the digestibility of CP, with a larger amino acid pool absorbed in the small intestine of sheep, explains the higher levels of total protein in the serum of sheep that received higher doses of WV (Buryakov et al., 2022). Additionally, the increasing availability of N–NH_3_ for absorption by the ruminal epithelium, with increased doses of WV, resulted in greater substrate for hepatic-urea synthesis and increased serum urea (Hanigan et al., 2018). On the other hand, the quadratic serum urea response may be associated with the increased hepatic transamination (see quadratic GGT elevation) associated with serum protein synthesis (see quadratic globulin elevation) (Hristov et al., 2019).

## 5 Conclusion

The supply of up to 40 mL d^−1^ of WV increases NDF intake, CP apparent digestibility, and N–NH_3_ in sheep rumens. We recommend offering up to 40 mL d^−1^ of WV to sheep fed high-concentrate diets. Studies with higher doses of WV are needed. Future studies may focus on evaluating the impact of using WV on different types of diets, growth performance, and the meat or milk quality of ruminants.

## Data Availability

No data sets were used in this article.
